# PTSD Among Healthcare Workers During the COVID-19 Outbreak: A Study Raises Concern for Non-medical Staff in Low-Risk Areas

**DOI:** 10.3389/fpsyt.2021.696200

**Published:** 2021-07-12

**Authors:** Ruike Zhang, Tianya Hou, Xiangyu Kong, Guibin Wang, Hao Wang, Shuyu Xu, Jingzhou Xu, Jingwen He, Lei Xiao, Yajing Wang, Jing Du, Yujia Huang, Tong Su, Yunxiang Tang

**Affiliations:** ^1^Department of Medical Psychology, Naval Medical University, Shanghai, China; ^2^Department of Respiratory Medicine, Huoshenshan Hospital, Wuhan, China; ^3^Department of Neurology, The Second Affiliated Hospital of Hunan Normal University (The 921 Hospital of the Chinese PLA Joint Logistic Support Force), Changsha, China; ^4^Medical Psychology Department, 96609 Military Hospital, Yinchuan, China

**Keywords:** COVID-19, healthcare workers, post-traumatic stress disorder, sleep, avoidance, intrusion, hyperarousal

## Abstract

**Objective:** To investigate the prevalence of sleep quality and post-traumatic stress disorder (PTSD) symptoms of healthcare workers (HCWs) and identify the determinants for PTSD symptoms among HCWs in high-risk and low-risk areas during the COVID-19 outbreak in China.

**Methods:** The Pittsburgh Sleep Quality Index and the Impact of Event Scale were used to assess sleep quality and symptoms of PTSD of 421 Chinese HCWs, respectively, from January 30 to March 2, 2020. The influencing factors of PTSD symptoms were identified by univariate analysis and multiple regression.

**Results:** The incidence of HCWs getting PTSD symptoms were 13.2%. HCWs from high-risk areas had significantly poorer sleep quality (*p* < 0.001). Poor sleep quality was the risk factor of PTSD symptoms for HCWs from high-risk (*p* = 0.018) and low-risk areas (*p* < 0.001). Furthermore, non-medical staff were found to be the risk factor for PTSD symptoms only in low-risk areas.

**Discussion:** HCWs in Hubei had poorer sleep quality. Non-medical HCWs from low-risk areas were associated with more severe PTSD symptoms. Mental health programs should be considered for HCWs, especially those who are often overlooked.

## Introduction

Several pneumonia cases of unknown etiology were first detected in Wuhan, Hubei Province, in China at the end of 2019 and the World Health Organization (WHO), China Office, was informed in a timely manner and responded to the outbreak of Novel Corona-virus (COVID-19) on January 5, 2020 (1). The outbreak of COVID-19 went from being declared as a Public Health Emergency of International Concern (PHEIC) to a global pandemic on March 11 by WHO after the epidemic had widely spread to the rest of the world (2).

Compared with SARS-CoV and MERS-CoV, COVID-19 was less severe but more infectious, according to rapidly increasing incidence and evidence of human-to-human transmission (3).

Healthcare workers (HCWs) are those at risk of confronting outbreaks and pathogens unknown to date (4) and are at high risk of being infected (5–7). In light of the magnitude of the COVID-19 pandemic and the stress experienced by HCWs, intense researches have investigated the psychological impact of the HCWs during the pandemic. Research has shown that HCWs experienced higher psychological morbidity, especially high-risk HCWs (8–11). A study has found that even in areas where the epidemic was not so severe, the risk of infection for HCWs is still higher than that of general population (12). Some research focused on low-risk epidemic areas has found that the mental problems of post-traumatic stress symptoms (PTSS), depression and anxiety were also found both in high-risk and low-risk HCWs (13, 14).

Post-traumatic stress disorder (PTSD) is a trauma related disorder that is characterized by the presence of one of the four symptoms of intrusion, avoidance, negative mood, and cognitive changes, as well as arousal and reactivity, for at least 1 month (15). A recent study has found that emergency workers had a 3-fold higher risk of PTSD than the general population (16). Hubei was the region with the most severe pandemic, and working in Hubei province was considered to be working in a high-risk area in this study, and HCWs from low-risk areas were those working outside Hubei province (including Shanghai, Beijing, Shandong, Chongqing, etc.). A study found that there were no interregional differences of stress following the outbreak among HCWs between Hubei or non-Hubei areas (17). On the contrary, other research has found that working in the high-risk epidemic area of China, Wuhan, entailed higher risk psychological distress (18).

However, most of the previous literature regarding the psychological effects of the pandemic on HCWs has focused particularly on doctors, nurses, and physicians. In comparison, there has been little research exploring the impact of the pandemic on hospital logistics and administrative staff. Research has found that nurses had a greater risk of experiencing anxiety and PTSD symptoms than other healthcare workers (19). In contrast, other research has reported more severe anxiety, depression, and insomnia problems in non-medical staff and physician in trainee compared with professional physicians (20). A study has found that workers in administration departments have similar anxiety scores to those of workers in clinical departments and fever clinics (21). These studies suggested that non-medical workers have similar mental problems with medical HCWs during the pandemic. A limited number of studies have investigated the PTSD symptoms in non-medical staff during the COVID-19 pandemic. A study has investigated the associations between the psychological health and physical symptoms among healthcare workers including doctors, nurses, administrators, and maintenance workers; however, it did not examine the psychological differences between medical HCWs and non-medical HCWs (22). Research has found that there was no significant difference in the detection rate of SARS-CoV-2 transmission in hospital in non-clinical HCWs compared with clinical HCWs (23), since non-medical HCWs were in hospital settings with less medical expertise. There is a very great demand for a study of the PTSD symptoms in medical as well as non-medical HCWs.

A systematic review of risk factors for PTSD among HCWs during pandemics has found that the position at work, level of exposure, quarantine, work experience, gender, and marital status were associated with PTSD (24). However, no previous study has investigated the different determinants of PTSD development between HCWs from high-risk and low-risk areas during the pandemic. Mental health, especially PTSD, could have a profound impact on the healthcare system. Therefore, it is necessary to investigate the prevalence of mental health disturbances and the risk factors of PTSD among HCWs during COVID-19, and specific interventions should be designed targeting those who are vulnerable to the development of PTSD during the outbreak. In this study, we assessed the prevalence of PTSD symptoms and their associated factors. We sought to analyze the predictive effect of demographic variables on PTSD symptoms in HCWs from high-risk and low-risk areas during the epidemic. As COVID-19 continues to spread over the next few months, this research may help identify HCWs who are more vulnerable to develop PTSD, which may provide a basis for further intervention.

## Methods and Materials

### Study Design and Participants

This study was a quantitative survey using the snowball sampling strategy for HCWs in China, including doctors, nurses, medical technicians, and non-medical staff working in hospitals. The time span of the study was 33 days between January 30 and March 2, 2020. A total of 421 HCWs voluntarily participated and completed questionnaires anonymously online. Participants over 18 years of age and who can read and understand Mandarin were included, while the exclusion criteria were being outside of China and time in bed less than actual sleep time. The study was approved by the Research Ethics Commission of Naval Medical University.

### Measures

Sociodemographic variables were collected, such as gender, age, years working, education level, marital status, occupation, being the only child of the family, and child status. The HCWs were divided into two categories according to their occupation: medical healthcare workers (i.e., doctors, nurses, medical technicians) and non-medical healthcare workers (i.e., administrative staff, logistical staff, and others).

The Pittsburgh Sleep Quality Index (PSQI) was used to test HCWs' sleep quality. It is a self-administered scale including 19 items consisting of seven dimensions including subjective sleep quality, sleep latency, sleep duration, sleep efficiency, sleep disturbances, use of sleep medications, and daytime dysfunction. The PSQI global score ranges from 0 to 21 (25). Scores >7 indicate poor sleep quality.

The Impact of Event Scale (IES-R) was used to assess subjective stress caused by traumatic events. The IES-R scale includes 22 items and consists of three subscales: intrusiveness, avoidance and hyperarousal. The scale ranges from 0 to 88. An IES-R total score >33 is identified as having PTSD symptoms (26).

### Statistical Analysis

PTSD total score were converted to dichotomous variables (presence of PTSD symptoms and no PTSD symptoms). The group comparisons of categorical variables were carried out with chi-square tests, and continuous variables were analyzed with Student's *t*-test. For univariate analysis of PTSD symptoms, the chi-square test was used for categorical variables. The count and frequency were presented.

Variables with *p* < 0.2 in univariate analysis were subjected to multiple regression analysis (27). Multiple logistic regression analysis using the forward conditional procedure was conducted for detecting risk factors for PTSD symptoms. The IES-R subscale scores were not normally distributed. Therefore, the Kruskal-Wallis test was used to compare the IES-R subscale scores across the different occupational groups and between HCWs from high-risk and low-risk areas.

A two-sided *p* < 0.05 was identified as statistically significant. All statistical analyses were performed using SPSS 26.0 (Statistical Package for the Social Sciences) for Windows (SPSS, Chicago, IL).

## Results

### Baseline Information

Of the 421 respondents completed the questionnaire, 401 participants were included in the study (response rate = 95.2%). The Expectation Maximization (EM) interpolation method was used to fill in the missing values. Most of the participants were distributed in Hubei, where the epidemic was the most serious across the country. Thus, HCWs in Hubei were considered HCWs from a high-risk area. Females account for 69.1% of the participants. The occupation of the HCWs in this study were classified into medical HCWs (i.e., doctors, nurses, and medical technicians) (*n* = 351, 87.5%) and non-medical HCWs (*n* = 50, 12.5%) including logistic and administrative staffs and others. Most of the participants were between 31 and 40 years old (*n* = 180, 44.9%) and have more than 10 years of work experience (*n* = 176, 43.9%). About 40% of the participants had poor sleep quality (*n* = 166, 41.4%). The data showed that the prevalence of PTSD symptoms was 13.2% (Table 1).

**Table 1:** Sociodemographic variables of HCWs.

**Characteristics**	***N* or Mean**	**Frequency or SD**
**Hubei Province**		
Non-hubei	232	57.9%
Hubei	169	42.1%
**Gender**		
Male	124	30.9%
Female	277	69.1%
**Occupation**		
Medical HCWs	351	87.5%
Non-medical HCWs	50	12.5%
**Education**		
Associate	66	16.5%
Bachelor's	265	66.1%
Master's/Doctorate	70	17.5%
**Only child in one's family**		
Yes	134	33.4%
No	267	66.6%
**Marital status**		
Married	278	69.3%
Other	123	30.7%
**Child status**		
No child	139	34.7%
Have children	262	65.3%
**Age**		
≤ 30	124	30.9%
31–40	180	44.9%
>40	97	20.2%
**Years of working**		
≤ 10	176	43.9%
11–20	131	32.7%
>20	94	23.4%
**Sleep quality**		
Normal sleep quality	235	58.6%
Poor sleep quality	166	41.4%
**PTSD**		
Yes	53	13.2%
No	348	86.8%

### Differences Between HCWS From High-Risk and Low-Risk Areas

We tested the differences of PSQI, PTSD symptoms and component scores between groups of high-risk area and low-risk area. The difference in sleep quality between HCWs from high-risk and low-risk areas is significant (*p* < 0.001). HCWs from high-risk areas had poorer sleep quality. The scores in high-risk area groups were significantly higher in subjective sleep quality (*t* = −3,365, *p* = 0.001), sleep duration (*t* = −6.425, *p* < 0.001), habitual sleep efficiency (*t* = −2.072, *p* = 0.039), sleep disturbances (*t* = −2.308, *p* = 0.022), use of sleep medications (*t* = −2.275, *p* = 0.024) and daytime dysfunction (*t* = −3.176, *p* = 0.002). The difference of PTSD symptoms (*p* = 0.690) was not significant between high-risk areas (*n* = 21, 12.4%) and low-risk areas (*n* = 32, 13.8%) HCWs. However, the intrusion scores were significantly different between HCWs from high-risk and low-risk areas (*p* = 0.041) (Table 2).

**Table 2:** Comparisons of the IES-R scores obtained by HCWs from high-risk area and HCWs from low-risk area.

**Variable**	**HCWs from high-risk area**	**HCWs from low-risk area**	***p***
PTSD symptoms	21 (12.4%)	32 (13.8%)	0.690
Hyperarousal	5.25 ± 3.934	4.99 ± 4.365	0.257
Intrusion	8.10 ± 5.270	7.18 ± 5.429	0.041^*^
Avoidance	5.67 ± 4.801	5.53 ± 5.021	0.603

*IES-R, The Impact of Event Scale (IES-R) scale was used to assess subjective stress caused by traumatic events. IES-R total score > 33 is identified to have PTSD symptoms*.

*HCWs, Healthcare workers; PTSD, Post-traumatic stress disorder*.

* *p < 0.05*.

### Comparisons of PTSD and Sleep Quality Between Medical and Non-medical HCWs

The prevalence of PTSD symptoms of medical HCWs and non-medical HCWs are 11.4 and 26.0%, respectively. The chi-square test showed that the PTSD symptoms were significantly different across occupational groups (*p* = 0.004). The differences between avoidance (*p* = 0.630), hyperarousal (*p* = 0.543) and intrusion (*p* = 0.672) were not significant across occupational groups (Table 3).

**Table 3:** Comparisons of the IES-R scores between medical and non-medical HCWs.

**Group**	**Medical HCWs**	**Non-medical HCWs**	***p***
PTSD symptoms	40 (11.4%)	13 (26.0%)	0.004^**^
Hyperarousal	4.98 ± 3.973	5.94 ± 5.423	0.543
Intrusion	7.48 ± 5.256	8.16 ± 6.172	0.672
Avoidance	5.54 ± 4.879	5.96 ± 5.268	0.630

*IES-R, The Impact of Event Scale (IES-R) scale was used to assess subjective stress caused by traumatic events. IES-R total score >33 is identified to have PTSD symptoms*.

*PTSD, Post-traumatic stress disorder*.

** *p < 0.01*.

### Risk Factors for PTSD Symptoms

Univariate analysis of influencing factors for PTSD symptoms showed that the variables of occupation (*p* = 0.004), marital status (*p* = 0.045), child status (*p* = 0.048), and sleep quality (*p* < 0.001) were significantly associated with PTSD symptoms (Table 4). The results of regression showed that for all HCWs, being a medical HCW (OR = 0.285, *p* = 0.002) was a protective factor for PTSD. Being married (OR = 2.453, *p* = 0.023) and having poor sleep quality (OR = 5.695, *p* < 0.001) were risk factors for PTSD. HCWs from high-risk and low-risk areas were used as stratification factors to explore further whether working in high-risk and low-risk areas would affect the risk factors for PTSD in HCWs. The risk factors for low-risk area HCWs were poor sleep quality and being non-medical HCWs. It should be noted that for high-risk area HCWs, the risk factor for PTSD was poor sleep quality (OR = 3.968, *p* = 0.018), which was different from that of HCWs from low-risk areas (Table 5). The multiple linear regression showed that poor sleep quality was a risk factor for intrusion (*p* < 0.001) and avoidance (*p* < 0.001). The risk factors for hyperarousal were poor sleep quality (*p* < 0.001) and being non-medical staff (*p* = 0.046) (Table 6).

**Table 4:** Univariate analysis of influence factors of PTSD symptoms.

	**PTSD**		
**Characteristics**	**Yes**	**No**	***p***
**Categorical variable**			
Hubei Province			0.690
Non-Hubei	32 (60.4)	200 (57.5)	
Hubei	21 (39.6)	148 (42.5)	
Gender			0.446
Male	14 (26.4)	110 (31.6)	
Female	39 (73.6)	238 (68.4)	
Occupation			0.004^*^
Medical HCWs	40 (75.5)	311 (89.4)	
Non-medical HCWs	13 (24.5)	37(10.6)	
Education			0.814
Associate	10 (18.9)	56 (16.1)	
Bachelor's	35 (66.0)	230 (66.1)	
Master's/Doctorate	8 (15.1)	62 (17.8)	
Only child in one's family			0.593
Yes	16 (30.2)	118 (33.9)	
No	37 (69.8)	230 (66.1)	
Marital status			0.045^*^
Married	43 (81.1)	235 (67.5)	
Other	10 (18.9)	113 (32.5)	
Child status			0.048^*^
No child	12 (22.6)	127 (36.5)	
Have children	41 (77.4)	221 (63.5)	
Age			0.118
≤ 30	10 (18.9)	114 (32.8)	
31–40	27 (50.9)	153 (44.0)	
>40	16 (30.2)	81 (23.3)	
Years of working			0.130
≤ 10	17 (32.1)	159 (45.7)	
11–20	19 (35.8)	112 (32.2)	
>20	17 (32.1)	77 (22.1)	
Sleep quality			<0.001^***^
Poor sleep quality	40 (75.5)	126 (36.2)	
Normal sleep quality	13 (24.5)	222 (63.8)	

* *p < 0.05*,

*** *p < 0.001*.

**Table 5:** Multiple logistic regression of risk factors of PTSD symptoms.

**Variable**	**All^a^**			**High-risk area^b^**			**Low-risk area^c^**		
**PTSD**	**OR**	**(95%CI)**	***p***	**OR**	**(95%CI)**	***p***	**OR**	**(95%CI)**	***p***
Age			NS			NS			NS
≤ 30									
31–40									
>40			ref			ref			ref
Occupation						NS			
Medical HCWs	0.285	(0.129, 0.625)	0.002^**^				0.239	(0.098, 0.581)	0.002^**^
Non-medical HCWs			ref			ref			ref
Years of working			NS			NS			NS
≤ 10									
11–20									
>20			ref			ref			ref
Child status			NS			NS			NS
No child									
Have children			ref			ref			ref
Sleep quality									
Poor sleep quality	5.695	(2.890. 11.220)	<0.001^***^	3.968	(1.262, 12.478)	0.018^*^	7.078	(3.004, 16.673)	<0.001^***^
Normal sleep quality			ref			ref			ref
Marital status						NS			NS
Married	2.453	(1.133, 5.310)	0.023^*^						
Other			ref			ref			ref
Constant	–2.493			1.423			–1.719		
Nagelkerke R square	0.186			0.133			0.250		

*PTSD, Post-traumatic stress disorder; NS, Not selected for adjusted logistic regression model; Ref, reference*.

*Dependent variable:*

a *PTSD syndrome for all HCWs*,

b *PTSD syndrome for HCWs from high-risk area*,

c *PTSD syndrome for HCWs from low-risk area*.

*Predictive variables tested by Forward: conditional method: Age, Occupation, Years of working, Marital status, Child Status, Sleep quality*.

* *p < 0.05*,

** *p < 0.01*,

*** *p < 0.001*.

**Table 6:** Multiple linear regression to predict PTSD symptoms.

**Variable**	**Standardized beta**	**B (95% CI)**	***p***
**Avoidance**		
**Sleep quality**		
Normal sleep quality	1.0	ref	
Poor sleep quality	0.221	2.207 (1.213, 3.200)	<0.001^***^
**Gender**		
Male	1.0	ref	
Female	0.113	1.198 (0.108, 2.288)	0.031^*^
**Intrusion**		
**Sleep quality**		
Normal sleep quality	1.0	ref	
Poor sleep quality	0.336	3.660 (2.617, 4.703)	<0.001^***^
**Hyperarousal**		
**Sleep quality**		
Normal sleep quality	1.0	ref	
Poor sleep quality	0.462	3.919 (3.148, 4.690)	<0.001^***^
**Occupation**		
Medical HCWs	1.0	ref	
Non-medical staff	0.098	1.240 (0.022, 2.457)	0.046^*^

*PTSD, Post-traumatic stress disorder*.

*Predictive variables tested by Enter method: Age, Occupation, Years of working, Marital status, Child Status, Sleep quality, Hubei province, Gender, Education and Only child in one's family*.

* *p < 0.05*,

*** *p < 0.001*.

## Discussion

This study investigated the prevalence of sleep quality and mental disturbances of HCWs during the pandemic and presented the potential influence value of demographic characteristics. In all, 13.2% of HCWs were shown to have PTSD symptoms in this study. The rates were lower compared with a meta-analysis that showed that 20.2% of the medical staff endured serious post-traumatic stress symptoms during and shortly after the epidemic (10). This is perhaps due to the different sample sources.

During the COVID-19 outbreak, HCWs in Hubei province had significantly poorer sleep quality (*p* < 0.001). When it comes to the component scores of the PSQI, the study showed that HCWs in Hubei has worse subjective sleep quality, shorter sleep duration, more sleep disturbances, worse sleep efficiency, more frequent use of sleep medications, and more severe daytime dysfunction. This finding was consistent with that of Grainne M. McAlonan et al. (28), who found that high-risk HCWs had a higher risk of fatigue and having poor sleep quality during the outbreak of SARS. Hubei Province was the center of the epidemic, where the number of confirmed cases and severe cases was significantly higher than those in other provinces. Therefore, the workload and work intensity of medical personnel in Hubei province were much greater than those in other provinces. And the HCWs working in Hubei province during this period are considered HCWs from a high-risk area with a much higher probability of being infected, which means particular attention should be paid to this group.

This study found that HCWs working in or outside Hubei province had an equal level of incidence of psychological stress. Recent research on 526 nurses found that PTSD symptoms were more severe in second-line nurses compared with that of frontline nurses (29). Other literature on the mental health of 994 HCWs in Wuhan has reported that exposure to the virus significantly increases the odds of PTSD symptoms (30). Previous studies have also indicated that similar psychological morbidity and perceived stress were found between high-risk and low-risk HCWs (31). COVID-19 has a long incubation period and the virus carriers are undetectable and could transmit virus during the latent period. Although non-Hubei provinces had a lower incidence than Hubei province, the number of confirmed cases was rising rapidly as well. The HCWs outside Hubei province did not recognize themselves as exempt from the danger. Moreover, the medical staff in Hubei might have had higher vigilance and confidence as the attention of the whole country was focused on them. Thus, medical staff in Hubei had a morale and sense of responsibility to conquer this challenge, which is beneficial for the maintenance of their mental health.

Our study has suggested that non-medical staff, such as administrative and logistic staff and others working outside Hubei province, had a higher incidence of PTSD symptoms compared with that of medical staff. Our findings match earlier observations. The attack rate among HCWs during SARS varied by occupations. The attack rate of HCWs in Vietnam, in 2003, were 16%, 35% for doctors and nurses, respectively. Those with the highest attack rate were administrative staff and “other staff with patient contact,” which accounted for 55% of cases (1). A cross-sectional study of 5,657 individuals showed that nonmedical staff endured a higher risk of depression, anxiety, and insomnia (32). This might be one of the reasons why administrative and logistic staff in our study endured a higher incidence of PTSD symptoms. Another reason could be that the logistic staff and others working in hospital might not be as psychologically prepared as doctors and nurses. Moreover, non-medical staff were not as well aware of the hallmark symptoms, precautionary measures, and hygiene issues, nor did they have the same prevention requirements as medical staff. Research showed that perception of higher risk (33) and lack of professional training (34) were the main occupational factors associated with PTSD. These suggested that the mental well-being of non-medical HCWs warrants more attention. The whole country was shut down, and the greatest responsibility of disease prevention fell to administrative staff. They were burdened with the consequences of clusters of infection, which are unpredictable. The administrative and logistic staff were faced with great pressure from both the upper authorities and the conditions of the epidemic. They were under tremendous pressure, both psychologically and physically. The tasks were arduous. Furthermore, situations of low exposure can also carry the risk of getting affected (35), as there were asymptomatic carriers. Non-medical staff in low-risk areas are easily neglected in an outbreak. We should be more concerned about their mental health.

However, the situation was different for HCWs from high-risk areas, for whom non-medical staff were no longer a risk factor for PTSD symptoms. Almost all the HCWs in Hubei province were drawn from other parts of the country. Hospitals in Hubei, like Huoshenshan and Leishenshan, have fixed management processes. All HCWs were concentrated in designated hospitals, their daily lives and protection requirements were exactly the same, and the division of labor was not that different. Their psychological conditions tend to be similar.

Our data showed that the risk of having PTSD symptoms, including avoidance, intrusion, and hyperarousal, tended to increase with poor sleep quality among HCWs. Research showed that sleep problems can affect the development of PTSD and the severity of symptoms (36). In this case, sleep disturbances are likely to be the risk factor and consequence of PTSD symptoms. Therefore, we could deal with mental health problems by coping with sleep disturbances, which are less stigmatizing.

The study has several limitations. First, it is a cross-sectional study, which cannot investigate the causal relationship. Second, there is self-report bias because all results were from self-reported questionnaires. Besides, setting up a true control group is impossible for our study, for all the HCWs in China were influenced by the COVID-19 outbreak. And we didn't recruit non-health care workers as a control group in this study. In addition, the study was conducted shortly after the outbreak of COVID-19, and the sleep quality and psychological problems of HCWs may not be really reflected in the survey. In addition, the sample of the study is relatively small, which might influence the generalization of the results. Finally, there might be selection bias that could also influence our findings.

## Conclusion

Our study indicated the predictors of PTSD symptoms among HCWs during the early stages of COVID-19. The study found that those often neglected, such as non-medical HCWs from low-risk areas, were at high risk for PTSD symptoms. We hope the results will be helpful for psychological professionals and policymakers in developing specific policies and mental health advice for HCWs, especially those with specific characteristics.

## Data Availability Statement

The datasets presented in this study can be found in online repositories. The names of the repository/repositories and accession number(s) can be found in the article/Supplementary Material.

## Ethics Statement

The studies involving human participants were reviewed and approved by Research Ethics Commission of Naval Medical University. The patients/participants provided their written informed consent to participate in this study.

## Author Contributions

RZ participated in conception, design of the work, data interpretation and analysis, drafting, and revision of the manuscript. TH, XK, JH, and LX participated in the acquisition and interpretation of data. GW, HW, SX, JD, YW, YH, and JX participated in the data analysis and revision of the draft. YT and TS made contributions to the concept and design of the study, acquisition of data, manuscript revision, and supervision. All authors approved the publication of this final version.

## Conflict of Interest

The authors declare that the research was conducted in the absence of any commercial or financial relationships that could be construed as a potential conflict of interest.

## Abbreviations

HCWs: Health care workers
PTSD: Post-Traumatic Stress Disorder
PSQI: The Pittsburgh Sleep Quality Index
IES-R: The Impact of Event Scale
PHEIC: Public Health Emergency of International Concern
WHO: World Health Organization.
