# Supplementary material for: PTSD Among Healthcare Workers During the COVID-19 Outbreak: A Study Raises Concern for Non-medical Staff in Low-Risk Areas
Source: Front Psychiatry. 2021 Jul 12;12:696200. doi: 10.3389/fpsyt.2021.696200 (PMC8310947; doi:10.3389/fpsyt.2021.696200)
Supplement: Supplementary file 1 [file Data_Sheet_1.PDF]

We have chosen to deposit the data to openICPSR.

The doi of the research data is https://doi.org/10.3886/E119159V1.
